# A deep learning model for prediction of autism status using whole-exome sequencing data

**DOI:** 10.1371/journal.pcbi.1012468

**Published:** 2024-11-08

**Authors:** Qing Wu, Eric M. Morrow, Ece D. Gamsiz Uzun

**Affiliations:** 1 Department of Molecular Biology, Cell Biology and Biochemistry, Brown University, Providence, Rhode Island, United States of America; 2 Center for Translational Neuroscience, Robert J. and Nancy D. Carney Institute for Brain Science and Brown Institute for Translational Science, Brown University, Providence, Rhode Island, United States of America; 3 Center for Computational Molecular Biology, Brown University, Providence, Rhode Island, United States of America; 4 Developmental Disorders Genetics Research Program, Department of Psychiatry and Human Behavior, Emma Pendleton Bradley Hospital, East Providence, Rhode Island, United States of America; 5 Department of Pathology and Laboratory Medicine, Warren Alpert Medical School of Brown University, Providence, Rhode Island, United States of America; 6 Department of Pathology and Laboratory Medicine, Rhode Island Hospital, Providence, Rhode Island, United States of America; Universita degli Studi di Torino, ITALY

## Abstract

Autism is a developmental disability. Research demonstrated that children with autism benefit from early diagnosis and early intervention. Genetic factors are considered major contributors to the development of autism. Machine learning (ML), including deep learning (DL), has been evaluated in phenotype prediction, but this method has been limited in its application to autism. We developed a DL model, the Separate Translated Autism Research Neural Network (STAR-NN) model to predict autism status. The model was trained and tested using whole exome sequencing data from 43,203 individuals (16,809 individuals with autism and 26,394 non-autistic controls). Polygenic scores from common variants and the aggregated count of rare variants on genes were used as input. In STAR-NN, protein truncating variants, possibly damaging missense variants and mild effect missense variants on the same gene were separated at the input level and merged to one gene node. In this way, rare variants with different level of pathogenic effects were treated separately. We further validated the performance of STAR-NN using an independent dataset, including 13,827 individuals with autism and 14,052 non-autistic controls. STAR-NN achieved a modest ROC-AUC of 0.7319 on the testing dataset and 0.7302 on the independent dataset. STAR-NN outperformed other traditional ML models. Gene Ontology analysis on the selected gene features showed an enrichment for potentially informative pathways including calcium ion transport.

## Author summary

Autism is a developmental disability. Genetic factors are considered as major contributors to the development of autism. Here, we present a deep neural network model, Separate Translated Autism Research Neural Network (STAR-NN), to predict autism status using WES data. STAR-NN showed a modest performance and was validated using an independent dataset. However, ML based models may have advantages in autism status prediction using genomic data and should be further studied.

## Introduction

Autism is a developmental disability that affects social and communication skills. In recent years, autism has been identified among 1% of children in the world population [1]. Studies have found that early diagnosis of autism was significantly associated with improved social characteristics [2]. To date, there is evidence supporting the importance of early diagnosis, followed by early intervention for having long-term positive impact [3–6]. While autism is highly heterogeneous, genetic variations are major contributors to the development of autism [7–11]. Rare genetic variants have garnered substantial focus [10,12]. Hundreds of loci and genes associated with autism have been identified in recent years with the advancement of next generation sequencing (NGS) [7,8,10,12–15]. Clinical genetic testing, including whole exome sequencing (WES), to identify rare variants is recommended [5–8].

Machine learning (ML) methods, including advanced deep learning (DL) models, offer the advantage of capturing subtle patterns among input features, making them efficient tools for phenotype prediction and feature weight inference [16]. Liu et al. developed a DL model to identify structural variations associated with several brain conditions, including ADHD, depression, and autism using whole genome sequencing (WGS) datasets as input [17]. DL methods have been used in cancer research for various purposes, ranging from predicting disease status (primary or metastatic) to prognosis prediction [18–22]. Both ML models and a limited number of DL models have been tested for autism prediction and the identification of autism subgroups [23–28]. As the diagnosis of autism is based on the observation of phenotypic presentations, DL models that used medically observed features, such as those from electronic health records (EHR), achieved a good prediction rate. Onishchenko et al. trained a DL model using EHR data from 30 million children under 6-years old to assess the comorbidity of autism [23]. Lin et al. trained a random forest and support vector machine classifier on gene expression profile from 31 children with autism to identify subgroups [25]. Using WES data from 598 schizophrenia families and 2392 autism families, researchers used the Extreme Gradient Boosting (XGBoost) model to classify individuals with autism and individuals with schizophrenia achieving a prediction accuracy above 80% [26]. However, these ML studies which used genetic data as an input lack an independent dataset to demonstrate their predictive ability and to further test the applicability of their method beyond the scope of training and testing data. Previous studies also found that small datasets might exhibit ascertainment bias in data collection. Successful biomedical ML models trained and tested on small datasets often failed on large heterogeneous datasets [29,30].

In this study, we developed a DL model to predict autism status using WES and microarray data. The model was trained and tested using a WES dataset, which includes 43,203 individuals (16,809 individuals with autism and 26,394 non-autistic controls). We further validated the model performance using an extra independent dataset from 27,879 individuals (13,827 individuals with autism and 14,052 non-autistic controls). Our model took into account the effects of rare and common variants [10,12,14]. A total of 1489 features, including a binary gender status, polygenic score (PGS) and 1487 genes, were pre-selected as the key features for our model.

## Results

### Autism status prediction model with selected features

**Fig 1:**
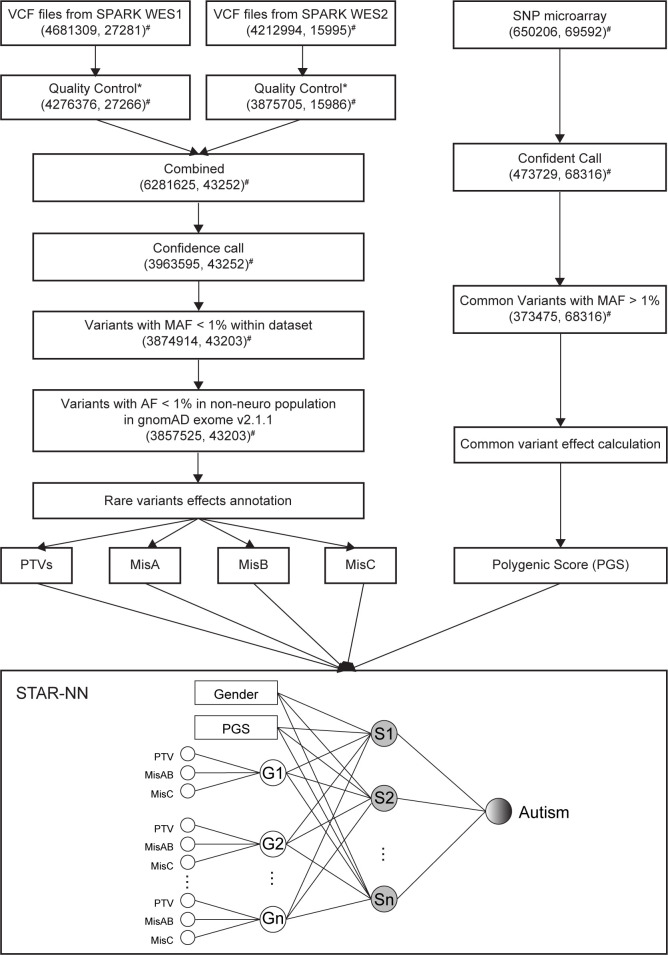
The workflow and framework of STAR-NN. After quality control, rare variants (minor allele frequency, MAF < 1%) identified from whole exome sequencing data were separated into four categories based on their function effect: protein truncating variants (PTVs), MisA (Missense variants with MPC > 2), MisB (Missense variants with 1 < MPC < 2) and MisC (Missense variants with 0 < MPC < 1). MisA and MisB were then combined as MisAB. Three types of rare exonic variants were used as input for STAR-NN model. In addition, polygenic score (PGS) generated from common variants (MAF > 1%) from microarray data were also used as input for STAR-NN. STAR-NN uses a three-to-one mapping strategy to learn different types of variants on the same gene separately. G represents gene node, S with grey color represents the option to add gene sets node before final output (shaded circle). *, Quality control on WES1 and WES2 used the same standards, further details provided in Materials and Methods. #, numbers in brackets showing (the count of variants, in the count of individuals) in the dataset.

We developed a DL model to predict autism status based on the individuals’ genetic data (Fig 1). We named our model “Separate Translated Autism Research–Neural Network (STAR-NN)” as it separates variants based on their functional effects on each gene. The impact of both common and rare variants was considered in our model. Rare protein truncating variants (PTVs) include nonsense, frameshift and canonical splicing variants. Missense variants were annotated by Missense Badness, Polyphen-2, Constraint (MPC) score [31] and separated into three groups: MisA, MisB and MisC (Methods). A higher MPC score indicates a higher likelihood of damaging effect of a missense variant. Missense variants with MPC score above 1, predicted as possibly damaging missense variants, were grouped into MisA and MisB forming MisAB. MisC represents the possibly benign missense variants. Due to the specificity of model structure, STAR-NN learned the impact of different types of variants in the same gene separately. Gender, PGS calculated from significantly associated common variants (MAF > 1%), together with three types of rare variants, PTVs, MisAB, and MisC, were used as input to predict a binary autism status: individuals with autism or non-autistic controls. We trained and tested the model using the combined WES1 and WES2 datasets (WES12) from SPARK dataset (16,809 individuals with autism and 26,394 non-autistic controls). 80% of the samples were used for training, 10% for validation and 10% for testing.

While selecting features is not a necessary step in DL models, the DL model might not efficiently learn the importance of certain rare features due to the sparsity of the rare variants. For that reason, we used an automated ML model, Tree-based Pipeline Optimization Tool (TPOT) [32], to select the best features for the model (Methods, S1 Fig). A final list of 1489 selected features, including 1487 genes, PGS and gender were used. Of the 1487 pre-selected genes (S1 Table), 115 genes have previously been found in “SFARI Gene”, a database of genes implicated in autism susceptibility [33] (hypergeometric test, p-value = 3.057e-5. SFARI genes list released on 1/11/2022).

**Fig 2:**
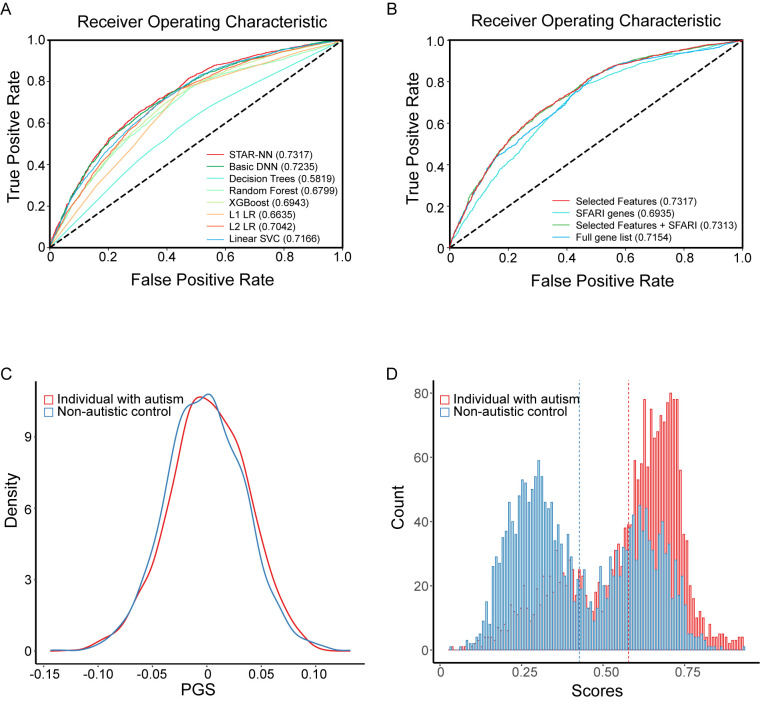
Performance of STAR-NN. **A**. ROC-AUC plot, showing STAR-NN outperformed six traditional machine learning model and a basic deep neural network (DNN) model. Variants of different type was not separated in traditional machine learning model and the basic DNN. **B**. ROC-AUC plot, showing STAR-NN with selected gene features outperformed the model using other gene sets as input. **C**. The density plot of PGS for individuals with autism and non-autistic controls. **D**. The distribution plot of score generated from STAR-NN for individuals with autism and non-autistic controls.

Our model outperformed traditional ML models, including decision trees, random forest, XGBoost, L1 logistic regression, L2 logistic regression, linear support vector and a basic DNN model that does not separate the rare variants by their functional effect, further supporting the importance of separation of variants based on their functional effect. Receiver Operating Characteristic Area Under the Curve (ROC-AUC) for STAR-NN was 0.7317 (Fig 2A and S2 Table). Our model demonstrated a faster training process than logistic regression with L2 regularization (L2LR) which exhibited the highest performance among the traditional ML models. STAR-NN took an average of 179.4 seconds for each training process whereas L2LR took an average of 690 seconds for each training (S3 Table). We used 1 CPU, 32 core and 256G memory for the training of each model on high performance computing cluster, Oscar maintained and supported by Center for Computation and Visualization at Brown University.

To test the predictive performance of selected features, we compared the model performance using 4 different groups of gene features as input, including 1487 selected gene features, 1031 SFARI genes, a combination of 2405 selected features and SFARI genes and the 19117 full gene set. We found that our model, using selected features has the highest performance (ROC-AUC = 0.7317) (Fig 2B and S4 Table).

**Fig 3:**
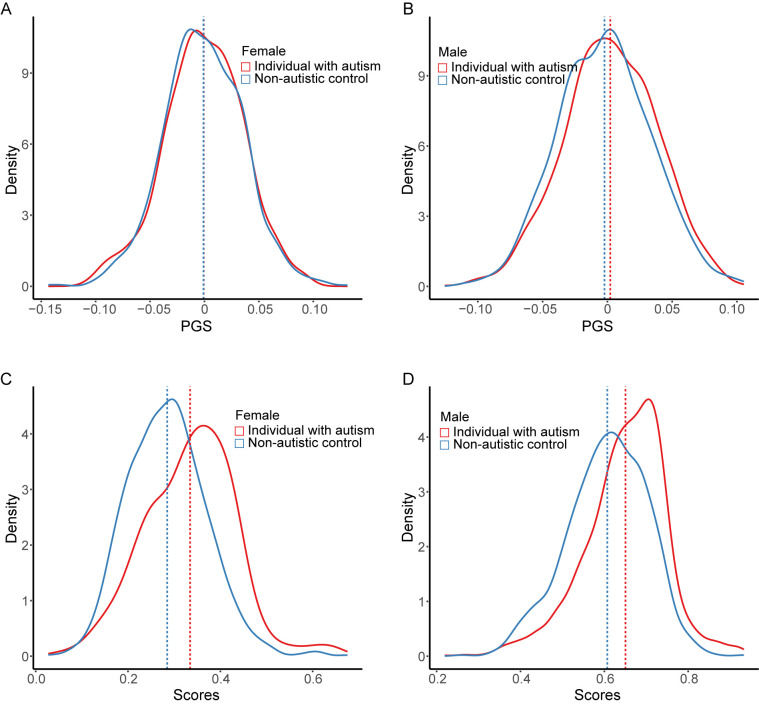
Score from STAR-NN in male and female population. The density plot of PGS for individuals with autism and non-autistic controls in females (**A**) and males(**B**). The density plot of autism score generated from STAR-NN in females (**C**) and males (**D**). The dashed line shows the mean value for each distribution.

We generated PGS using common variants for each individual (Fig 2C). Compared to the PGS, which exhibits a small difference between individuals with autism and non-autistic controls (mean PGS: 0.00156 for individuals with autism and -0.00152 for non-autistic controls), our model significantly separated the two groups (mean scores: 0.5784 for individuals with autism and 0.4271 non-autistic controls, respectively; Mann-Whitney-Wilcoxon Test, p<2.2e-16, Fig 2D). While a significant difference was observed between PGS of males with autism and non-autistic controls, PGS of females with autism and non-autistic controls shows no significant difference (Fig 3A and 3B). We also tested the STAR-NN model without PGS as an input and ROC-AUC was 0.7281 with gender, PTVs, MisAB and MisC as input (S5 Table). Compared to PGS, the score generated from STAR-NN has a significant distinction between individuals with autism and non-autistic controls for both males and females (Fig 3C and 3D).

We tested the individual effect of PTV, MisAB and MisC on the prediction of autism status. Basic DNN model using PGS, biological sex and aggregated count of PTVs per gene as input resulted in an ROC-AUC of 0.7080. The ROC-AUC of 0.7015 was generated using PGS, biological sex and aggregated count of MisAB per gene as input. The basic DNN model using PGS, biological sex and aggregated count of MisC per gene as input had a lower performance, (ROC-AUC of 0.6982) compared with using aggregated count of PTVs per gene or using aggregated count of MisAB per gene as input (S6 Table). We also tested STAR-NN with a 2-to-1 mapping structure to assess the necessity of including MisC as input. We used gender, PGS, aggregated counts of PTVs and MisAB as input and obtained an ROC-AUC of 0.7157 for the 2-to-1 STAR-NN model (S7 Table). Meanwhile, we tested STAR-NN model performance of a 4-to-1 mapping structure by separating PTVs, MisAB, MisC and synonymous variants on the same gene at the input level and merged into one. We found that including synonymous variants slightly decreased model performance (S8 Table). Each ROC-AUC value mentioned above were based on 10 random repeats. The results showed that PTV, MisAB and MisC are all contributing to the prediction of autism status. STAR-NN, incorporating the combined effect from PTVs, MisAB and MisC, had a slightly better performance than basic DNN model with individual effects of PTV, MisAB and MisC as input. This suggests the importance of 3-to-1 mapping structure of STAR-NN to the prediction of autism status.

### Validation with an independent dataset

To evaluate the predictive ability of STAR-NN model, we used an extra independent dataset which was also generated by SPARK. 27,879 participants were included in the most recent release of iWES1_v1 from SPARK (13,827 individuals with autism and 14,052 non-autistic controls). STAR-NN achieved similar performance with selected features and SFARI genes (ROC-AUC = 0.7302 for selected features, and ROC-AUC = 0.7319 for SFARI genes, S9 Table). The model performance using the independent dataset was similar to that using the original dataset.

### Input features additively contributed to the autism status prediction

In STAR-NN, we used sigmoid function in the output layer for autism status prediction. Sigmoid ensures a non-linear relationship between input features and the prediction results. Subsequently, we tested if there is a non-additive effect across input features. Deep neural network (DNN) model with multiple hidden layers captures the higher interactions across input features [20,34]. Therefore, we compared the performance of STAR-NN with no hidden layer, which models an additive contribution of gene features, to STAR-NN with one hidden layer, which models a higher interaction of biological pathways across input features. As an increase in model complexity might cause overfitting problems and thereby reduce model performance, we also tested the STAR-NN with one hidden layer using “relu” and “linear” as the activation function, separately. Both showed similar performance in prediction accuracy, precision and recall rate (ROC-AUC = 0.7285 for “relu” as activation function, ROC-AUC = 0.7284 for “linear” activation function) (S10 Table) [35]. Therefore, we kept the structure of STAR-NN with no hidden layer between gene layer and the output layer, indicating the gene features are additively contributed to the autism status prediction.

### Genes related to developmental disorders were enriched in selected genes

To investigate the function of selected genes, we performed a disease ontology pathway enrichment analysis. We found that the selected genes are enriched with genes related to developmental disorders, intellectual disability and autism (S2 Fig). Then, we performed a gene ontology (GO) term analysis to study the biological function of selected genes. GO terms associated with stimulus, including “detection to stimulus” and “response to stimulus” were significantly enriched. Synapse and dendrite related pathways, including “postsynapse”, “dendrite” and “glutamatergic synapse” were significantly enriched. “Ion binding” and “ion transport” related GO terms were also significantly enriched (S12 Table). The counts of variants in selected features are provided in supplementary tables (S11 Table). Previous findings showed that pathways related to ion channel, including calcium ion channel, potassium ion channel and sodium ion channel are significantly associated with autism [36–38].

## Discussion

Autism is a developmental disability with variability in phenotypic features and genetic characteristics. Autism is highly heritable, from 64% to 91% [39]. Rare genetic variants have been identified as major contributors [10,12]. In a clinical setting, patients are advised to undergo genetic testing to evaluate the presence of rare variants [40–43]. With the advances in NGS technologies and bioinformatics tools, we need better tools to interpret complex genetic test results. Applications of ML methods have been evaluated in precision medicine research. SVMs and RF were the top two most commonly used ML algorithms in studies on phenotype prediction and factor identification [25,30,44–47]. Compared to traditional ML methods, DL models as an advanced part of ML with more flexible model construction to capture non-linear and additive effect across input features have been studied in clinical phenotype and subtype classification, such as primary and metastatic cancer classification [19–21]. To date, limited studies have used either traditional ML or DL models in autism status prediction [23,25,26,28]. The constraints for usage of genetic data for autism status prediction include 1) lack of large datasets to support the effectiveness of developed model in a heterogeneous population, and 2) lack of an external dataset to prove the predictive ability of developed models. Small datasets might have the potential risk of ascertainment bias, which increases the uncertainty of model performance in a heterogeneous setting [30]. An external dataset is needed to test the reproducibility of the model results outside the scope of training and testing dataset.

Recent studies showed that variants with different level of damaging effects on the same gene contribute differently to the development of autism [7,8,48]. PTVs and possibly damaging missense variants (MisAB) were found to be significantly enriched in individuals with autism and individuals with developmental delay (DD), while mild effect missense variants (MisC) were found to be enriched in DDs only [7,48,49]. In STAR-NN, by constructing a 3-to-1 sparse connection between variants layer and gene layer, the model learned the effect of different level of damaging variants on the same gene separately. Besides, STAR-NN also considered PGS which generated from common variants [8]. In this way, STAR-NN predicted autism status with a modest ROC-AUC of 0.73 and a F1 score of 0.69.

With the expectation to improve the prediction accuracy by capturing higher interaction, the non-additive effect, across gene features, we also tested the model performance by adding hidden layers between the gene layer and the output layer. However, the best performance was still obtained from the model with single gene layer, suggesting the pre-selected gene features, PGS and gender worked in a linear additive way to the prediction of autism status with current sample size. This finding is aligned with the results in a recent study [49], in which the higher interaction across sex, PGS and rare variants score were tested using pairwise linear model and only found the additive effect across input features. As the performance of deep neural network is affected by sample sizes, more samples are needed to test the existence of higher interactions across input features.

In STAR-NN, we used aggregated count of variants on genes as input, considering the impact of variants on a gene level to the prediction of autism status. A similar inference method was also used in P-NET and BANNs, both of which are biologically interpretated neural network models [20,50]. In an alternative approach, Jiao et al. used aggregated variants detected by WGS per 1M bp bin as input for their DL model to classify primary and metastatic cancer [21]. In our study, we selected 1487 gene features. SHANK3 is a well-known autism gene and in SFARI database. GOPC gene was previously found among the top significantly differentially expressed genes in a Parkinson’s disease iPSC model and was identified as a potential target in the treatment of Parkinson [51,52]. Despite autism is a neurodevelopmental disorder, studies also found some parkinsonism features, such as motor issues, in older adults with autism [53,54]. TYW3 gene has been previously found associated with amyotrophic lateral sclerosis (ALS) and tic spectrum disorder (TSD) [55,56].

Copy number variants (CNVs) are genetic factors important for the etiology of autism. To date, 71 autism loci have been identified from CNV and WES data [12]. Gene expression profiles generated from a small dataset with autism patients have previously been used to predict subgroups in which RF and SVM were applied [25]. Besides mutation data, CNVs, gene expression and DNA methylation should be considered in ML models, as previous study suggested a connection between impaired methylation and the etiology of autism [57,58]. To further improve the prediction accuracy in the future, these data types should be included when available. Larger sample size is also needed to better detect the higher interactions across genetic features. Another limitation in STAR-NN is the population used for training and testing the model. Around 70% of the individuals in WES12 are from European population. More samples from diverse populations are needed to further train and test the model. Overall, STAR-NN showed a modest performance to predict autism status. Further studies are needed to assess the viability of ML based models to predict autism status using genomic data.

### Ethics approval and consent to participate

The research performed in the study is approved by institutional review board (IRB) of Brown University.

## Materials and methods

### SPARK whole exome sequencing (WES) data

The sequencing was performed by Simons Foundation Powering Autism Research for Knowledge (SPARK) [59,60]. Variants were called by GATK v4.2.1.0 [61], weCall v2.0.0 [62] and DeepVariant v1.2.0 [63,64] separately and stored in the SPARK genomic dataset. While both GATK and weCall reported a higher number of variants than DeepVariant, DeepVariant has been documented as more precise compared to other callers [63,65]. Studies showed that the number of false positive variants called by GATK was higher compared to those called by DeepVariant [65,66]. Thus, in this study we utilized vcf files produced by DeepVariant. The WES1 and WES2 datasets were released in September 2019 and June 2020, respectively. The WES1 and WES2 cohorts are 77.12% and 73.86% European, respectively. The population distributions are shown in a histogram in S3 Fig. From the 27,281 participants in WES1, 4,681,309 variants were called. From the 15,995 participants in WES2, 4,212,994 variants were called.

An additional independent dataset was also generated by SPARK. This dataset was released in February 2022, two years after the release of the WES1 and WES2 datasets. A total of 27,879 participants were included comprising13,827 individuals with autism and 14,052 non-autistic controls. In this dataset, 8,336,937 variants were called by DeepVariant. However, the SPARK datasets may contain related individuals and therefore kinship is not accounted for in the model.

### Quality control

Hail (version 0.2.99) was used to perform quality control on 4,681,309 variants from 27,281 participants in WES1 and 4,212,994 variants from 15,995 participants in WES2. Initially, variants on low complexity regions were removed. Variants labeled as “MONOALLELIC” were also removed. The remaining number of variants were 4,537,198 and 4,085,509 in WES1 and WES2, respectively. Subsequently, we excluded genotypes with the following criteria: 1) genotype calls on chromosome Y for female participants; 2) genotypes with read depth lower than 10 or higher than 1000.; 3) for homozygous reference calls, genotype with the allele balance higher than 0.1 with genotype quality lower than 25; 4) for homozygous alternative calls, genotype with allele balance lower than 0.9 with the phred-scaled likelihood (PL) lower than 25 for homozygous reference; 5) for heterozygous or hemizygous calls, genotypes with allele balance lower than 0.25 and PL for heterozygous reference lower than 25. Samples with a call rate lower than 90%, average genotype quality lower than 20 and average genotype depth lower than 10 were excluded. In WES1 and WES2, 27,266 and 15,986 samples remained, respectively. Regarding variant QC, variants with a call rate above 0.1 and Hardy-Weinberg equilibrium p-value greater than 1e-12 were retained. This resulted in 4,276,376 variants from the 27,266 participants in WES1 and 3,875,705 variants from the 15,986 participants in WES2. We merged the data from WES1 and WES2 incorporating a total of 6,281,625 variants from 43,203 participants in our study (excluding 91 duplicated samples).

### Rare variants and variant annotation

To obtain high confidence rare variants, variants with genotype quality lower than 25 or call rate lower than 90% were removed. Rare variants were defined as minor allele frequency (MAF) less than 1% among samples in SPARK dataset and MAF less than 1% in non-neuronal population in gnomAD (exome v2.1.1.). A total of 3,857,525 variants remained. To make our model work efficiently, synonymous variants were excluded, due to the CADD score of synonymous variants is relatively low compared with nonsense variants and missense variants (S4 Fig). 1,469,036 rare variants from 19,117 genes were kept for the model development. The above variants were annotated by dbNSFP4.2a using Annovar (version: 2018Apr16) [67]. We categorized missense variants by their Missense badness, Polyphen2, Constraint (MPC) score [31]. We utilized the three-tier classification of missense variants from the study by Satterstorm et al [7]. MisA represents missense variants with MPC score above 2, indicating the probably damaging missense variants group; MisB represents missense variants with MPC score between 1 and 2, indicating the possibly damaging missense variants; MisC represents missense variants with MPC score below 1, indicating mild impact missense variants.

### SPARK genotype data and common variants

Genotype data from 69,592 samples in the SPARK dataset were downloaded. PLINK 1.9 [68,69] was used to generate confidence common variant calls from 650,206 variants. 1,276 samples with more than 10% missing genotypes were excluded. 121,896 variants that failed the Hardy-Weinberg equilibrium exact test with a p-value<1e-06 were excluded. Common variants were defined as having a MAF above 1%. 100,254 variants were excluded due to a lower MAF. 373,475 common variants from 68,316 samples were retained. Subsequently, common variants for individuals from WES1 and WES2 rare variants data were matched using the unique individual identifier (SP_ID, provided in the data).

### Generation of gene by sample matrix

The genotype of rare variants in 43,227 samples have been converted to 0,1,2 representing homozygous reference, heterozygous and homozygous alternative, respectively. The matrix table of variants-by-sample was sparse (greater than 99%). We converted the variants-by-sample matrix into gene-by-sample matrix using: *G*_*ij*_ = ∑_*k*_ *V*_*kj*_, where *k* represents the number of variants in gene *i*. *V*_*kj*_ = 0 if sample *j* does not carry the variant, *V*_*kj*_ = 1 if sample *j* carries the variant. Therefore, *G*_*ij*_ represents the aggregated count of variants in gene *i* of sample *j*. In STAR-NN model, where PTVs, MisAB and MisC on the same gene were separated, the *G*_*ij*_ represents the aggregated count of single type of variant in gene *i* of sample *j*.

### Generation of PGS

Effect sizes of common variants were calculated using SBayesR [70]. Summary statistics, including variant ID, rsID (or the genomic location of variant when rsID is not provided), effect allele, alternative allele, odds ratio of effect allele, standard error of odds ratio, p-value, allele frequency of effect allele and per-variant sample size, generated by Grove et al. were used as input for SBayesR–gwas-summary parameter [8]. The banded LD matrix (https://cnsgenomics.com/software/gctb/#LDmatrices) provided by SBayesR were used as input for–mldm parameter. We kept the other parameters as default and excluded MHC region. The output of SBayesR were used as input of–score parameter for PGS calculation by PLINK 1.9.

### Feature selection

We employed an automated ML method, Tree-based Pipeline Optimization Tool (TPOT) [32], for the feature selection and the hyperparameter tuning for each feature selection method. Four feature selection methods from sklearn python package were used in TPOT, including “removing features with low variance”, “univariate feature selection”, “recursive feature elimination” and “feature selection using SelectFromModel”. Simultaneously, we generated four gene-by-sample matrices based on variant type (PTV, MisA, MisB and MisC) for feature selection. The value in the matrix is the aggregated count of variants per genes per sample in the training data, which comprises 80% of the samples. Feature selection in TPOT is performed on training data only. The differences across four matrices are the different combination of variants (S1 Fig).

Features selected using methods “SelectFwe” and “SelectionPercentile” from sklearn python package, had the highest ROC-AUC for each sample by gene matrix. In total, 954 genes from 19,117 genes were selected from sample-by-gene matrix covering aggregated count of PTVs and missense variants per gene. 350 genes from 16,832 genes were selected from sample-by-gene matrix covering aggregated count of PTVs per gene. 348 genes from 17,483 genes were selected from sample-by-gene matrix covering aggregated count of PTVs and deleterious missense variants (MisA) per gene. 365 genes were selected from 18,284 genes using sample-by gene-matrix covering aggregated count of PTVs, deleterious missense variants (MisA) and possibly deleterious missense variants (MisB) per gene (S1 Table). We combined selected genes from four matrices and removed overlaps resulting in 1487 selected genes.

### Gene ontology (GO) and disease ontology (DO) enrichment analysis

GO and DO enrichment analysis were performed on selected genes using gprofiler2/v0.2.1 R package [71] and DOSE/v3.22.1 R package [72], respectively.

### STAR-NN model

STAR-NN is a feedforward neural network with nodes representing variants and genes, and edges representing location relationship between variants and genes. In STAR-NN, rare variants were pre-annotated and separated into three categories: PTVs, possibly damaging missense variants (MisAB, a combination of MisA and MisB) and possibly benign missense variants (MisC). The input layer is the variant layer with each node representing the aggregated count of one type of variants in one gene (57,375 input nodes for 3 variant types from 19,125 genes). The second layer is the gene layer with each node representing a gene. The connection between variant layer and gene layer is sparse and follows a three-to-one pattern, in which variants are only connected to their located gene. For genes that do not carry certain type of variants, we used 0 to represent the blank. The connections between gene layer and output layer were fully connected. The output of each layer follows *f*(*x*) = *g*(*w*∙*x*+*b*), where *w* is the weight, *x* is input of each layer and *b* is bias, *g*() represents the “tanh” function, $\frac{\left(e^{2x}-1\right)}{\left(e^{2x}+1\right)}$, in sparse layer, “relu” function, $\left\{ \begin{array}{c} x,x>0\\ 0,x<0 \end{array}\right.$, in gene layer and “sigmoid” function, $\frac{1}{1+e^{-x}}$, in the output layer. The learning rate was initially set as 3*10^−5^ with a learning rate decay follows * $\frac{1}{1+\frac{10^{-5}}{30}*epoch}$, where *lr* represents initial learning rate. Adam optimizer was used to reduce binary cross entropy loss between the label of a training sample and the predicted probability output of the sample [73]. The model makes an early stop if the loss for validation dataset does not further decrease in 50 epochs and retrieves the model parameter from the best performance according to the minimum loss. The model outputs a probability score between 0 and 1. The prediction is made based on a 0.5 threshold, where score above 0.5 indicates autism and score below 0.5 indicates non-autistic control. The prediction performance is measured using metrics of average F1, ROC-AUC, binary accuracy, precision, and recall rate over 10 random repeats on one random split. The performance of model on 10 random repeats on 10 random splits showed a similar performance and the results are saved in S13 Table.

For the basic DNN model used as a baseline comparison, we did not separate variants in each gene by their functional effect. Therefore, there is no variant layer in the basic DNN model. The aggregated count of variants in genes are used directly as input gene layer. Then, the gene layer is fully connected to the output layer. Gene layer has the following structure: *f*(*x*) = *g*(*w*∙*x*+*b*), where *g*() represents “sigmoid” function, the same as STAR-NN. The difference between the basic DNN model and the STAR-NN model is an additional input variant layer. The gene layer in STAR-NN takes the weighted aggregated effect of three types of variants per gene whereas the gene layer in the basic DNN model takes the unweighted aggregated effect of three types of variants per gene.

We also compared the performance of STAR-NN with six traditional ML models, including three tree-based models; decision trees, random forest, XGBoost, three linear models; L1 and L2 logistic regression, linear support vector classifier (LinearSVC). Same as the basic DNN model, aggregated variants in each gene were used as input for the traditional ML models. For traditional ML models, we also separated the training and testing data into 80% and 20% using the same random seed as used in the STAR-NN. We used default parameters settings for DecisionTreeClassifier and RandomForestClassifier from sklearn python package [74] and the default parameter settings of XGBClassifier from xgboost package [75]. For LogisticRegression with L1 regularization and LogisticRegression with L2 regularization from sklearn python package, we set C = 0.001 with “saga” solver and max_iter = 2000. For LinearSVC from sklearn python package, we used C = 0.001 and penalty = “l2”.

## Supporting information

S1 Fig Workflow of selected features using TPOT from four gene sets. (EPS)

S2 Fig Enriched disease ontology (DO) term from selected gene features. developmental disorder, intellectual disability, and autism are among the top enriched DO terms.
(EPS)

S3 Fig Population distribution on individuals from WES1 and WES2 datasets. (EPS)

S4 Fig CADD score distribution on synonymous variants (**A**), exonic variants (**B**) and splicing variants (**C**). CADD score above 20 indicates top 1% of most deleterious effect. Majority of synonymous variants have a CADD score below 20, a density peak at score of 1 and score of 8. Majority of splicing variants have a CADD score above 20 and a density peak above score of 30.
(EPS)

S1 Table The list of 1489 selected features generated by TPOT. (CSV)

S2 Table Traditional machine learning model performance using prediction accuracy, precision, recall, ROC-AUC and F1. (CSV)

S3 Table The training time for STAR-NN model and the logistic regression model with L2 regularization (LRL2). (CSV)

S4 Table STAR-NN performance comparison on four gene sets in testing dataset. Four gene sets include selected features, SFARI genes, combination of SFARI genes and selected features and full gene list.
(CSV)

S5 Table STAR-NN performance on input without PGS. (CSV)

S6 Table STAR-NN performance on input from single type of variants. (CSV)

S7 Table STAR-NN performance on input without mild effect missense variants. (CSV)

S8 Table STAR-NN performance on input with additional synonymous variants. (CSV)

S9 Table STAR-NN performance comparison on four gene sets on extra independent dataset. (CSV)

S10 Table STAR-NN model performance comparison on 1) no additional hidden layer, 2) one additional hidden layer between the gene layer and the output layer using relu activation function and 3) one additional hidden layer between the gene layer and the output layer using linear activation function.
(CSV)

S11 Table Count and frequency of variants in four populations, including male cases, male controls, female cases, and female controls. Counts of variants represents the aggregated variants in each population group. Variant frequency is the count of variant divided by total number of individuals in each population group. Proportion of variants between cases and controls in male and female are also calculated. Proportion is calculated based on variant frequency in each population group.
(XLSX)

S12 Table Enriched GO terms on 1487 selected genes. (CSV)

S13 Table STAR-NN performance on 10 random splits on 10 random repeats. (CSV)
